# Update on Thrombocytopenia in Pregnancy

**DOI:** 10.1055/s-0040-1721350

**Published:** 2020-12-21

**Authors:** Simone Filipa Carrasqueira Subtil, Jorge Miguel Bastos Mendes, Ana Luísa Fialho de Amaral Areia, José Paulo Achando Silva Moura

**Affiliations:** 1 Universidade de Coimbra, Coimbra, Portugal

**Keywords:** thrombocytopenia, pregnancy, preeclampsia, HELLP syndrome, thrombotic microangiopathy, trombocitopenia, gravidez, pré-eclâmpsia, síndrome HELLP, microangiopatia trombótica

## Abstract

Thrombocytopenia, defined as platelet count < 150,000 mm ^3^ , is frequently diagnosed by obstetricians since this parameter is included in routine surveillance during pregnancy, with an incidence of between 7 and 12%. Therefore, decisions regarding subsequent examination and management are primordial. While most of the cases are due to physiological changes, as gestational thrombocytopenia, other causes can be related to severe conditions that can lead to fetal or maternal death. Differentiating these conditions might be challenging: they can be pregnancy-specific (pre-eclampsia/HELLP syndrome [hemolysis, elevated liver enzymes, low platelets]), or not (immune thrombocytopenia purpura, thrombotic thrombocytopenic purpura or hemolytic uremic syndrome). Understanding the mechanisms and recognition of symptoms and signs is essential to decide an adequate line of investigation. The severity of thrombocytopenia, its etiology and gestational age dictates different treatment regimens.

## Resumo

Trombocitopenia, definida como uma contagem de plaquetária < 150.000 mm ^3^ , é frequentemente diagnosticada pelos obstetras, uma vez que este parâmetro está incluído na vigilância de rotina durante a gravidez, com uma incidência de entre 7 e 12%. Assim, decisões relativas à avaliação e orientação subsequentes são primordiais. Embora a maioria dos casos ocorra devido a alterações fisiológicas, como a trombocitopenia gestacional, outras causas podem estar relacionadas com condições graves que podem levar à morte fetal ou materna. Distinguir entre estas entidades pode ser desafiante: elas podem ser específicas da gravidez (pré-eclâmpsia/síndrome HELLP [ *hemolysis, elevated liver enzymes, low* *platelets* ]) ou não (púrpura trombocitopênica imune, púrpura trombocitopênica trombótica ou síndrome hemolítico urêmico). Compreender os mecanismos e reconhecer os sinais e sintomas é essencial para decidir uma adequada linha de investigação. A severidade da trombocitopenia, a sua etiologia e a idade gestacional ditam regimes de tratamento diferentes.

## Introduction

Thrombocytopenia, defined as platelet count of < 150,000 mm ^3^ , is a frequent diagnosis during pregnancy, occurring in 7 to 12% of pregnancies. 1 2 Mild thrombocytopenia corresponds to platelet count > 100,000 mm ^3^ , moderate between 50,000 and 100,000 mm ^3^ , while severe thrombocytopenia relates to platelets < 50,000 mm ^3^ . 3 It may be related to physiologic changes or pathological conditions, some of which are unique to pregnancy and may pose significant risk to both mother and child. 1

Thrombocytopenia typically results in mucosal bleeding consequent to primary hemostasis defect. 1 4 As so, clinical presentation includes epistaxis, gingival bleeding or abnormal uterine bleeding; other common signs are petechiae and ecchymosis. Life-threatening bleeding is infrequent and is restricted to patients with extremely low platelet levels, presenting as hematuria, gastrointestinal bleeding and, rarely, intracranial hemorrhage. 1 However, platelet count > 50,000 mm ^3^ is mostly asymptomatic, if their function is normal. 3 In pregnancy, most cases of thrombocytopenia are due to hemodilution and increased platelet destruction. Decreased production is not common, and when it occurs, it is mainly associated with nutritional deficiencies. 1 A complete medical history including medication, medical conditions and physical exam are mandatory. Laboratory testing should include complete blood count, peripheral blood smear, liver and renal function tests, coagulation study, antiphospholipid antibodies, antinuclear antibodies, human immunodeficiency virus (HIV) serology, hepatitis C antibody and hepatitis B surface antigen. 2 5

### Physiological Changes during Pregnancy

It is usual to find low platelet count in pregnant women, beginning in the first trimester and gradually decreasing through gestation, with nadir at delivery. 6 This condition is due to physiological hemodilution, increased platelet activation and clearance, and transient flow sequester in placental circulation. 7 A recent retrospective cohort study of 4,568 women evaluated the course of platelet count throughout uncomplicated pregnancies. Comparing with the mean platelet count in nonpregnant women (273,000 mm ^3^ ), pregnant women had decreased count since the first trimester, worsening during gestation. Twin pregnancies had a lower platelet count during pregnancy and at delivery compared with single pregnancies, possibly due to greatest plasma volume or larger placental size. 6 8

### Pregnancy-Related Conditions

#### Gestational Thrombocytopenia

Gestational thrombocytopenia (GT) is the most common etiology of thrombocytopenia during gestation, occurring in 5 to 11% of pregnancies. 1 9 It is responsible for 75 to 80% of all thrombocytopenias in pregnant women, and its pathogenesis most likely results from hemodilution and accelerated platelet clearance. 1 3 9 10 There is no personal history of thrombocytopenia outside pregnancy, but the risk of recurrence is 14.2 times higher among women with previous GT. 1 6 11 Gestational thrombocytopenia can occur at any point of pregnancy, but it is most frequent during mid-to late-second or third trimester. 11 In the setting of thrombocytopenia in pregnancy, it is indicated a complete blood count and examination of the peripheral blood smear, to exclude pseudothrombocytopenia due to EDTA platelet clumping. 1 8 Stable platelet count > 100,000 mm ^3^ in asymptomatic pregnant women are due to GT and do not require further investigation nor specific intervention other than periodic monitoring. 1 3 8 9 12 There is no evidence of the ideal frequency of platelet count, but a monthly platelet count is suggested. This is a diagnosis of exclusion, with no specific laboratory tests. 1 Other causes should be considered if there is a platelet count < 100,000 mm ^3^ , as only between 1 and 5% of GT cases develop platelet count below this value (although some authors suggest investigation if platelet count is < 70,000 mm ^3^ ). 5 6 8 10 Most international guidelines suggest excluding epidural anesthesia with a platelet count between < 70,000 and 80,000 mm ^3^ , due to epidural hematoma risk and spinal cord compression; however, others accept lower platelets (50,000 to 70,000 mm ^3^ ), provided that there is no suspicion of pre-eclampsia (PE) or hemolysis, elevated liver enzymes and low platelets syndrome (HELLP syndrome). 1 8 12 An anesthetic evaluation during the third trimester of pregnancy is advised in cases of moderate to severe thrombocytopenia. 3 Steroids should be considered with platelets < 50,000 mm ^3^ , as the diagnosis of immune thrombocytopenic purpura (ITP) cannot be excluded; in GT, there is no response to steroids or intravenous immunoglobulin (IVIg). 3 12 Interventions as elective cesarean delivery are not indicated and there is no risk of maternal bleeding complications nor fetal hemorrhage, since this condition is not associated with neonatal thrombocytopenia). 1 3 5 Recovery to normal platelet count occurs within between 1 and 2 months of delivery, so a platelet count is recommended 6 weeks after birth. 3 10 12

#### Pre-eclampsia

Hypertensive disorders of pregnancy are responsible for 5 to 21% of maternal thrombocytopenia. 1 Pre-eclampsia is characterized by new onset hypertension after 20 weeks of pregnancy associated with at least one additional feature: proteinuria, thrombocytopenia (< 100,000 mm ^3^ ), renal insufficiency, impaired liver function, pulmonary edema and/or new onset of headache. 13 Nearly 50% of PE cases develop thrombocytopenia and it might be one of the earliest signs and preceding hypertension in PE, being considered a severe feature of this condition. 1 9 10 12 13 Typically, a sudden decrease in platelet count should lead to PE hypothesis. Hemorrhage seldom occurs, unless in the presence of disseminated intravascular coagulopathy (DIC). 1 The pathogenesis of thrombocytopenia in these cases is due to thrombotic microangiopathy (TMA), owing to injured endothelium causing platelet aggregation and adhesion, enhancing platelet consumption, and thrombin generation in small vessels. 3 9 12 Characteristic alterations include schistocytes on peripheral blood smear, elevated bilirubin and lactate dehydrogenase (LDH), with decreased haptoglobin. 12 Treatment is delivery, ideally at or after 34 weeks of gestation; expectant management of PE with severe features before 34 weeks depends on maternal and fetal stability and is based on strict selection criteria. 13 Platelet transfusions are reserved for patients with active bleeding or undergoing cesarean delivery, to increase maternal platelet count > 50,000 mm ^3^ . However, the benefit is temporary, due to accelerated platelet destruction. 1 Thrombocytopenia in the setting of PE or HELLP syndrome may improve more quickly with corticosteroids, but no differences were found in terms of maternal mortality or morbidity. 1 5 14 Decrease in platelet count is often seen for between 24 and 48 hours postpartum, improving rapidly thereafter, unlike other TMAs. 15

### Hemolysis, elevated liver enzymes and low platelets syndrome

Hemolysis, elevated liver enzymes and low platelets syndrome syndrome is characterized by platelet count < 100,000 mm ^3^ , elevated liver function tests and microangiophatic hemolytic anemia (MHA), related to endothelial damage and coagulation activation. 13 16 17 Hemolysis, elevated liver enzymes and low platelets syndrome may result from a continuum of PE, in 10% of cases, or might occur without pre-eclamptic features, like hypertension and proteinuria, in up to 15% of cases. 12 13 Besides being more frequent in the third trimester, it can present first postpartum, in 30% of the cases. 16 Clinical manifestations include upper abdominal pain, nausea, vomiting, malaise, headache and, rarely, jaundice. 17 This condition imposes an aggressive management, as DIC occurs in 20% of cases, which can lead to massive hemorrhage, placental abruption and hepatic rupture, implying high maternal morbidity and mortality. 3 17 Delivery is the only treatment, regardless of the gestational age. 13 Similarly to PE, there is insufficient evidence to support the use of corticosteroids in HELLP management, and 90% of patients will have platelet count > 100,000 mm ^3^ with supportive care within the first postpartum week. In case of DIC, fresh frozen plasma with or without cryoprecipitate may be required. 10 13 14 17

### Acute Fatty Liver of Pregnancy

This rare life-threatening condition (6–14:100,000 pregnancies) typically occurs in the third trimester and seems to be related to estrogen elevation in late pregnancy, fatty acid metabolism disorder and mitochondrial dysfunction. 18 Clinical manifestations include abdominal pain, malaise, anorexia, nausea and vomiting. As this condition progresses, liver failure and encephalopathy may ensue. Blood pressure usually is in the normal range. 8 17 Analytically, there is severe hypoglycemia, hyperuricemia, elevated transaminases and bilirubin, renal impairment, coagulopathy, and thrombocytopenia (present in < 50% of cases). 5 Patients can be diagnosed according to the Swansea criteria and acute fatty liver of pregnancy (AFLP) can be confirmed with hepatic biopsy, demonstrating microvesicular steatosis. 17 Severe HELLP syndrome is the main differential diagnosis, but hypoglycemia and coagulopathy are key features of AFLP. 10 17 Early delivery is mandatory, and supportive care may be required for several days or weeks, including correction of coagulopathy and dialysis. 17 Some authors suggest plasmapheresis, implying a mortality rate of 17%, compared with 81% in those with supportive care only. 18

### Medical Conditions

#### Primary Immune Thrombocytopenia

Primary immune thrombocytopenia, also known as ITP, is an acquired autoimmune disorder characterized by production of antiplatelet antibodies causing isolated thrombocytopenia, accounting for between 1 and 4% of pregnancy thrombocytopenia. 1 2 3 9 Impaired platelet production by thrombopoietin also plays a role. 3 4 Preconception or early pregnancy platelet count is important in distinguishing ITP from GT, since ITP typically presents prior to pregnancy or during the first trimester. 8 9 12 Similarly to GT, ITP diagnosis is based mainly on the exclusion of other causes of isolated thrombocytopenia, as definitive diagnostic tests are lacking. 1 8 Initial evaluation should include a complete medical history, full physical examination, full blood count and peripheral blood film, to exclude pseudothrombocytopenia or TMA ( Table 1 ). 7 Assays for the detection of antiplatelet antibodies lack both sensitivity and specificity, so they should not be performed. 1 3 Thrombocytopenia < 100,000 mm ^3^ is suggestive of ITP, and < 50,000 mm ^3^ is almost definitely related to this condition. 1 An international consensus suggests that pregnant patients with a suspicious history of ITP or with a platelet count < 80,000 mm ^3^ should be investigated for possible ITP. 7 Owing to transplacental passage of maternal IgG antiplatelet antibodies, there is a risk of fetal and neonatal thrombocytopenia. Although maternal platelet count does not predict fetal platelet count, there are some warning factors: a previously affected sibling is the strongest predictor of neonatal thrombocytopenia; and maternal antiplatelet circulating antibodies are reversely correlated to neonatal platelet count. 2 8 19 A retrospective case study showed that almost one-fifth of infants will develop platelet count < 150,000 mm ^3^ . 20 Neonatal platelet count < 30,000 mm ^3^ occurs approximately in between 1 and 5% of newborns, but severe hemorrhagic complications like intracranial hemorrhage are rare (< 1%). There is no evidence that cesarean delivery reduces the risk of intracranial hemorrhage. 2 3 10 Pregnancy surveillance should be managed combining obstetric and hematology settings: expert opinion suggests every trimester assessment in asymptomatic women in remission; if platelet count is < 80,000 mm ^3^ , weekly monitoring should be considered, especially after 34 weeks. 1 2 10 Usually, no treatment is required during pregnancy, since most cases present with platelet count > 70,000 mm ^3^ ; however, at least between 15 and 35% may require treatment before labor. 3 10 Most women do not have bleeding complications, although there is a slight increase in postpartum hemorrhage, especially if platelet count is < 20,000 mm ^3^ . 8 12 In fact, in a cohort study, 21% of women with severe thrombocytopenia had postpartum bleeding. 21 Accordingly, clinical practice guidelines from the American Society of Hematology advise treatment until 36 weeks or sooner if: delivery is imminent, or if platelet count falls < 30,000 mm ^3^ ; < 50,000 mm ^3^ near delivery; or if the patient is symptomatic. 5 Vaginal delivery is considered safe between > 20,000 and 30,000 mm ^3^ ; and operative vaginal or cesarean delivery with 50,000 mm ^3^ . So, before delivery, the platelet count should be maintained > at 50,000 mm ^3^ . 3 8 12 If ITP is suspected, fetal scalp electrodes and operative vaginal delivery such as vacuum or forceps should be avoided. 2

**Table 1:** Recommended tests x Associated conditions

Recommended tests	Associated conditions
CBC and peripheral blood smear	Pseudothrombocytopenia, pancytopenia, hemolysis
Reticulocyte count	Hemolysis, hypertensive disorders
Coagulation screening (PT/PTT) and fibrinogen	DIC, severe liver disease
Liver function	Preeclampsia, HELLP syndrome
LDH	Hemolysis
Viral serologies (HIV,HCV)	Viral infection
Renal function	HUS, TTP, DIC
**Optional**	
ANA/APS	Systemic lupus erythematosus, antiphospholipid syndrome
ADAMTS13 activity & antibody	TTP
H. pylori testing	H. pylori infection
Thyroid function	Thyroid disorders
Immunoglobulin levels	Immunodeficiency disorders

Abbreviations: ANA, anti-nuclear antibodies; APS, antiphospholipid antibodies; CBC, complete blood count; DIC, disseminated intravascular coagulopathy; HELLP, hemolysis, elevated liver enzymes, low platelets; HUS, hemolytic uremic syndrome; LDH, lactate dehydrogenase; PTT, partial thromboplastin time; PT, prothrombin time; TTP, thrombotic thrombocytopenia purpura.

If the maternal count is < 80,000 mm ^3^ in labor, a cord sample should be collected to check the baby's count, since it is difficult to exclude ITP. Until the fetal count is known, intramuscular vitamin K should be postponed. 3 8 Prednisolone and IVIg are considered first-line options. 5 In a retrospective study from two tertiary hospitals, there was no significant difference in maternal platelet response between the two. 22 Treatment should start with prednisolone, starting with 20 mg daily, increasing to 60 mg if there is an inadequate response in 1 week; the steroid dose should be tapered to the lowest effective dosage. 3 7 Some international guidelines recommend an initial dose of 1 mg/Kg, but there is no evidence that higher doses are more effective. 5 8 Platelet response is usually seen in between 4 and 14 days, and the same corticosteroid dosage should be maintained for 21 days before tapering. 1 2 Toward being able to receive epidural anesthesia, prednisolone can be initiated 10 days prior to anticipated delivery with between 10 and 20 mg/day in women with platelet count < 80,000 mm ^3^ . 5 In cases of inadequate response to steroids or if a rapid response is needed, IVIg has a rapid effect in increasing platelet count in between 1 and 3 days and a peak response within between 2 and 7 days. It is recommended 1 g/Kg as a one-time dose, and retreatment may be required every 2 to 4 weeks; increase in platelet count usually lasts 1 to 3 weeks. If there is no response, the next-line option is a combined prednisolone and IVIg treatment. IV immunoglobulin G anti-RhD treatment experience is limited. 1 2 3 7 8 Second-line treatments might be needed in women with platelet count < 20,000 to 30,000 mm ^3^ or < 50,000 mm ^3^ near delivery, besides steroid or IVIg therapy. Medication used in nonpregnant women lack safety data in pregnancy. Immunosuppressants as azathioprine and cyclosporine have been used in pregnancy with acceptable side effects, but with delayed response, 3 to 6 months. Rituximab's response occurs in between 1 and 8 weeks, and can be considered in severe cases. 7 8 Splenectomy can be considered, preferably during the second trimester, as first trimester surgery is linked to risk of miscarriage and surgery during third trimester is technically difficult. Romiplostin and eltrombopag, thrombopoietin-receptor agonists, lack safety data in pregnancy, so their routine use during pregnancy is not yet recommended. 8 Nevertheless, Rodriguez Nuñez et al. 23 reported nine ITP cases treated during pregnancy with romiplostin, which was considered as an alternative option, without serious maternal or fetal side effects; nonetheless, the majority of pregnant women were treated after 20 weeks. Eltrombopag has also been safely used during pregnancy. 24 Platelet transfusions are not routinely recommended, as they will not provide long-term response, but should be available in the setting of significant bleeding or if platelet count is < 50,000 mm ^3^ near delivery, in combination with IVIg. 2 5 8 Each unit of platelet concentration is expected to increase platelet count by 7,000 to 10.000 mm ^3^ in 1 hour in a 75-Kg individual. 2 Platelet count will not improve spontaneously after birth, unlike GT. 12 Nonsteroidal anti-inflammatory drugs (NSAIDs) should be avoided during the postpartum period since there is an elevated hemorrhagic risk. 7

#### Thrombotic Microangiopathies

Thrombotic microangiopathy can be pregnancy-related, such as preeclampsia and HELLP, or rarely nonpregnancy-related (for which pregnancy acts as a trigger), like thrombotic thrombocytopenia purpura (TTP) and hemolytic uremic syndrome (HUS). Both are characterized by endothelial damage, microvascular thrombosis, consumptive thrombocytopenia, and intravascular hemolytic anemia. 15 Unlike pregnancy-related TMA, which rarely occurs before the second trimester, TTP/HUS can present at any point during pregnancy and postpartum. In TTP, the mainstream form of presentation occurs after 30 weeks of pregnancy; HUS presents mostly postpartum. 25 26 27 In the setting of severe thrombocytopenia, even when considering preeclampsia or HELLP syndrome, an hemolysis screening including LDH levels and blood film should be requested. 17 In pregnancy with TMA, the stillbirth rate is high, ∼ 40%, caused by microvascular thrombosis, placental ischemia and infarction. 12 It is recommended to check regularly for signs of fetal distress or intrauterine growth restriction with uterine artery Doppler ultrasound. Premature delivery may be appropriate due to fetal prognosis. 12 17 Unlike PE/HELLP syndrome, the TTP/HUS disease course is not influenced by delivery. 12 15 Clinical persistence or deterioration between 48 and 72 hours postpartum should prompt TTP/HUS as a possible diagnosis, since PE/HELLP features improves within this period. 10 17

#### Thrombotic Thrombocytopenia Purpura

Thrombotic thrombocytopenia purpura is a rare (1 in 25,000 pregnancies) life-threatening blood disorder, in which microthrombi develop in small blood vessels due to lack of ADAMTS13 enzyme activity, leading to persistence of ultra-large multimeters of von Willebrand factor, activating platelet receptors and consequently platelet aggregation. 3 15 17 The characteristic pentad includes MHA, thrombocytopenia, neurological symptoms/signs, renal dysfunction and fever. 3 15 However, up to 35% of patients lack some of the typical signs. Neurological symptoms may include several manifestations from headache to coma. 12 Mutations in the *ADAMTS13* gene are responsible for congenital deficiency of von Willebrand factor-cleaving protein (Upshaw-Schulman Syndrome). More frequently, an acquired form occurs due to autoantibody production that blocks enzyme activity. These two different etiologies can be distinguished by the presence of the inhibitor. 3 The diagnosis of TTP may be confirmed by reduced ADAMT13 activity (< 10%) and/or by the presence of IgG antibodies to ADAMTS13. Additionally, laboratory findings include evidence of MHA, normal coagulation tests (TTP/HUS are not associated with coagulopathy) and increased serum creatinine. 12 15 17

Plasmapheresis allows antibody removal in the acquired form. Fresh frozen plasma should be infused until platelet count is restored and the LDH level is reduced. Immunosuppression is also required in immune-mediated TTP, with steroids and azathioprine. Monoclonal antibody against CD20 (rituximab) is considered standard of care in the general population, but during pregnancy it is usually initiated postpartum, for safety issues. In congenital forms, fresh frozen plasma is enough to augment ADAMTS13 levels. 3 15 Once platelet count is restored, the frequency of plasmapheresis or plasma infusion will depend on subsequent platelet count and ADAMTS13 activity levels. 17 Platelet transfusions may precipitate central nervous system (CNS) symptoms, so they are contraindicated. 3 15 Pregnancy may trigger both acquired and congenital TTP. The risk of acquired TTP recurrence is reported to be ∼ 50%, and the risk of congenital TTP relapse is 100%, if no prophylaxis measures are taken, as reported in a Japanese series. 26 28 Some authors suggest low dose aspirin associated with prophylactic low molecular weight heparin in high thrombotic risk women during pregnancy. 17 This approach, including regular plasma therapy in congenital TTP, has been associated with 100% live births and maternal survival. 26 In congenital cases, if term is achieved, delivery is advisable by 37 weeks and induction of labor and vaginal delivery is encouraged. 17 In the acquired type, ADAMTS13 activity should be monitored in early pregnancy and at least each trimester; if the ADAMTS13 activity falls to < 10%, plasma therapy with regular plasmapheresis and azathioprine as a steroid-sparing treatment should be considered. Rituximab can be used before conception to normalize ADAMTS13 levels in acquired cases. 17 26

#### Hemolytic Uremic Syndrome

The clinical presentation of HUS is similar to that of TTP (MHA and thrombocytopenia), but with a poor renal prognosis, since the risk of end-stage renal disease is between 44 and 55%. 27 Infection is the main cause during childhood, due to Shiga-toxin production by E.coli O157:H7. 29 The atypical form is linked to a congenital defect that results in dysregulation of the alternate complement system pathway, known as complement-mediated HUS (CM-HUS). 12 15 17 Indeed, pregnancy is a potent complement activator, accounting for 7% of CM-HUS cases. 30

This is an exclusion diagnosis, after ruling out TTP as well as other TMA causes. 15 17 Complement genetic testing can be performed to support the diagnosis. 25 Initial therapy includes plasmapheresis and fresh frozen plasma, and dialysis is frequently necessary. 12 Plasmapheresis is often ineffective, since the risk of end-stage renal disease is similar between patients who underwent plasmapheresis and those who did not, as shown by Bruel et al. 27 Eculizumab, a monoclonal anti-C5 inhibitor, is approved for CM-HUS in nonpregnant women and has been safely used in paroxysmal nocturne hemoglobinuria during pregnancy. 31 This complement inhibitor treatment is promising, since renal recovery may be achieved if eculizumab is started early. So, if CM-HUS is highly suspected, eculizumab should be started promptly. 15 27 A recent Russian series reports 35% maternal and 25% fetal mortality in CM-HUS. 30 Fetal outcomes seem better than in TTP, possibly because most cases present postpartum. 15 The risk of relapse and of chronic kidney disease should be discussed in women with history of CM-HUS, and a recommended approach is to restart eculizumab at the first sign of relapse. 15 Other authors suggest to start eculizumab in the second trimester, until the postpartum period, in women who are not already on complement inhibition therapy, as it can be difficult to anticipate relapse by routine laboratory tests in time to avoid serious maternal and fetal complications. 17

#### Secondary Immune Thrombocytopenia and Systemic Conditions

Viral infections can cause transient thrombocytopenia. Human immunodeficiency virus (HIV) and cytomegalovirus are frequent underlying agents, and thrombocytopenia is thought to be caused through antibodies cross-reacting with platelets. 2 3

Several medications can cause drug-induced thrombocytopenia, including unfractionated heparin (incidence of between 3 and 6%), penicillin, cephalosporins and NSAIDs. 2 3 A careful medication history is crucial for diagnosis. Antiphospholipid syndrome (APS) is an autoimmune disease characterized by arterial and venous recurrent thrombosis and/or recurrent early pregnancy loss, fetal loss or pregnancy morbidity related to placental insufficiency or pre-eclampsia. 32 Thrombocytopenia is a frequent clinical manifestation (20–40% of APS cases), mostly > 70,000 mm ^3^ . 33 Catastrophic APS, a rare acute multiorgan failure due to small vessels occlusion, may include thrombocytopenia in 60% of cases. Triggers are identified in 50% of cases, such as infections, surgery or obstetrics morbidity. 32 33 Pregnancy may also be a risk for acute “flares” of systemic lupus erythematosus, which might be associated to thrombocytopenia. 5 Disseminated intravascular coagulopathy implies an activation of the coagulation system leading to microvascular thrombus and multiple organ failure, mainly in the setting of placental abruption, uterine rupture, amniotic embolus and PE/HELLP syndrome. There will be thrombocytopenia, increase of the prothrombin time, partial thromboplastin time, decreased fibrinogen and increased fibrin degradation products like D-dimers. 12 Disseminated intravascular coagulopathy requires aggressive management, including fresh frozen plasma, cryoprecipitate and platelet transfusion. 3 Primary bone marrow disorders, such as myelodysplastic syndrome and acute leukemia, are extremely rare, but should be considered in the setting of pancytopenia, after exclusion of more frequent diagnosis such as nutritional deficiencies (folate or B12). 2 8 Vitamin B12 plays a vital role in DNA synthesis, and hematopoietic precursor cells are extremely sensitive to B12 deficiency. B12 deficiency should be suspected in the setting of prior bariatric surgery, inflammatory bowel disease, *Helicobacter pylori* infection, use of metformin or proton pump inhibitors, vegan diets or pernicious anemia. 34

### Heritable Platelets Function Disorders

#### Bernard-Soulier Syndrome

This autosomal recessive disorder affects < 1:1,000,000 individuals and is characterized by qualitative and quantitative defects of the platelet membrane glycoprotein Ib-IX-V complex. 9 35 Clinical manifestations include thrombocytopenia, prolonged bleeding time and the presence of giant platelets. Pregnancy course may be normal, but there might be severe bleeding during the peripartum period. 9 35 Primary postpartum bleeding has been reported in 33%, and secondary in 40% of pregnancies, leading in some cases to hysterectomy. Neonatal hemorrhagic complications such as intracranial bleeding may occur due to alloimmune thrombocytopenia. The use of rFVIIA associated with tranexamic acid is recommended, but DDAVP and platelet transfusions might be needed. The third stage of labor should be actively managed with uterotonics to avoid uterine atony. The safest mode of delivery is yet to be determined. 35

### Glanzmann Thrombasthenia

Glanzmann thrombasthenia is an autosomal recessive condition characterized by a deficiency or dysfunction of glycoprotein IIb-IIIa receptors on platelet, which is a receptor of fibrinogen, interfering with platelet aggregation, leading to prolonged bleeding time. 36 These individuals have normal platelet count and morphology, but there is no platelet aggregation. It is also associated with bleeding during pregnancy and the peripartum period. Different treatments have been proposed to prevent hemorrhage, such as platelet transfusion, recombinant factor VIIa concentrate and plasmapheresis, but due to the rarity of this disorder, recommendations are difficult to implement. Temporary fetal thrombocytopenia related to the presence of maternal HPA antibodies to platelet glycoproteins may lead to in utero death due to intracranial hemorrhage. There are no benefits in performing caesarean section to prevent neonatal morbidity/mortality. 9 36

## Conclusion

Gestational thrombocytopenia is by far the most common cause of thrombocytopenia during pregnancy and does not imply any fetal or maternal risk. The diagnosis of ITP, an immune-mediated condition, is one of exclusion; nevertheless, in almost two-thirds of cases there is a prepregnancy diagnosis. Most of the cases do not require treatment. Pre-eclampsia and HELLP syndrome are associated with thrombocytopenia, and early delivery may be necessary. Thrombotic microangiopathy due to TTP and CM-HUS occurs seldomly in pregnancy. Distinction from PE and HELLP syndrome may be challenging, and a multidisciplinary approach is often necessary. Intensive care management and close monitoring can help improve pregnancy outcomes. The prognosis for future pregnancies must be discussed with these women. The paucity of literature on the use of innovating effective agents in ITP and HUS (as thrombopoietin-receptor agonists or eculizumab), implies difficult decisions concerning risks of fetal and maternal outcomes associated with disease progression, against safety issues of their usage during pregnancy.
